# Neoadjuvant Chemotherapy Prior Fertility-Sparing Surgery in Women with FIGO 2018 Stage IB2 Cervical Cancer: A Systematic Review

**DOI:** 10.3390/cancers14030797

**Published:** 2022-02-04

**Authors:** Alessandro Buda, Martina Borghese, Andrea Puppo, Stefania Perotto, Antonia Novelli, Chiara Borghi, Elena Olearo, Elisa Tripodi, Alessandra Surace, Enrica Bar, Giovanni Scambia, Francesco Fanfani

**Affiliations:** 1 Division of Gynecologic Oncology, Michele e Pietro Ferrero Hospital, 12060 Verduno, Italy; stperotto@aslcn2.it (S.P.); cborghi@aslcn2.it (C.B.); etripodi@aslcn2.it (E.T.); asurace@aslcn2.it (A.S.); ebar@aslcn2.it (E.B.); 2 Clinic of Obstetrics and Gynecology, Santa Croce e Carle Hospital, 12100 Cuneo, Italy; borghese.m@ospedale.cuneo.it (M.B.); puppo.a@ospedale.cuneo.it (A.P.); antonia.novelli@aslcn1.it (A.N.); olearo.el@ospedale.cuneo.it (E.O.); 3 Dipartimento Della Salute Della Donna, del Bambino e di Sanità Pubblica, Fondazione Policlinico Universitario A. Gemelli IRCCS, 00168 Rome, Italy; giovanni.scambia@policlinicogemelli.it (G.S.); francesco.fanfani1@unicatt.it (F.F.); 4 Dipartimento Scienze Della Vita e Sanità Pubblica, Università Cattolica del Sacro Cuore, 00168 Rome, Italy

**Keywords:** fertility-sparing surgery, neoadjuvant chemotherapy, cervical cancer

## Abstract

**Simple Summary:**

In order to provide our contribution to the knowledge of women affected by IB2 cervical cancer, who wish to preserve fertility, we revised and updated the available literature in the debated issue of neoadjuvant chemotherapy prior to fertility-sparing treatment. The effectiveness of preoperative chemotherapy in tumors larger than 2 cm virtually proposes a conservative opportunity to a broader group of women, while keeping low, and hopefully absent, the risk of local and distant relapse. Available studies of neoadjuvant chemotherapy to the fertility-sparing approach have shown that a suboptimal response at surgery seems to be an independent prognostic factor in poorer survival, and, therefore, the careful selection of patients and the surgical approach after neoadjuvant chemotherapy remains crucial. Finally, we proposed an algorithm to be helpful in the decision-making process of that subgroup of patients.

**Abstract:**

Nowadays, the optimal management of patients with cervical cancers measuring 2–4 cm desiring to maintain fertility is still uncertain. In this systematic review, we assessed the reliability of neoadjuvant chemotherapy (NACT) prior to fertility-sparing (FS) surgery in International Federation of Gynecology and Obstetrics (FIGO) 2018 stage IB2 cervical cancer, in terms of pathologic response, oncological and obstetric outcomes. The review of the literature was performed following the Preferred Reporting Items for Systematic Reviews and Meta-Analyses (PRISMA) guidelines. Data, using MEDLINE and PubMed, were searched for from 1 January 2005 up to 1 December 2020. We identified 20 articles and 114 women with IB2 disease, possible candidates for NACT prior to FS surgery. However, uterine conservation was achieved only in 76.7% of them. Patients reached optimal pathological response to NACT in 60.9% of cases and a TIP (cisplatin, ifosfamide and paclitaxel) regime was related to the best response. Suboptimal response to NACT appeared to be an independent negative prognostic factor. Up to 9.2% of patients recurred with a median 7.4-months DFS, and 4.6% of patients died of disease. Fifty percent of women tried to conceive after treatment and NACT prior to conization appeared to be the most promising alternative to upfront radical trachelectomy in terms of obstetric outcomes. In conclusion, NACT prior to FS surgery is an option, but the literature about this issue is still weak and FS should be carefully discussed with patients.

## 1. Introduction

Cervical cancer is the second major cause of oncological death in women aged 20–39, and almost 40% of all cervical cancer diagnosis are made in this age frame. Nearly half of the time, the disease is apparently confined to the cervix [1]. Currently, the standard treatment for tumors measuring 2–4 cm in diameter is radical hysterectomy [2]; while radical trachelectomy, along with standard treatment, has been introduced for young women with tumors smaller than 2 cm desiring to maintain fertility [3]. An analysis of the SEER data (Surveillance, Epidemiology, and End Results) showed promising data regarding the oncological safety of uterine preserving surgery (conization/trachelectomy) when compared to radical treatment [4]. However, a tumor size larger than 2 cm was associated with a significantly worse outcome in patients undergoing conservative treatment, compared to radical hysterectomy. Other studies have also shown that the size of the tumor is one of the most relevant prognostic factors in this setting, increasing the risk of recurrence when lesions are greater than 2 cm [5,6].

Nowadays, optimal management of patients with tumors measuring 2–4 cm is still not clear as the available data is limited. Definitive radical hysterectomy is associated with a 13% risk of recurrence and a five-year disease-free survival rate of 87% [2]. A valid option for women desiring to maintain fertility is an upfront radical trachelectomy, even if the data has shown that a high proportion of patients might require adjuvant treatment, due to high-risk histological factors after the upfront surgery [7]. Moreover, obstetrical outcomes in patients appear to be unsatisfactory most of the time, with a frequent need for infertility treatments and with a high rate of second trimester abortions and preterm delivery, secondary to cervical incompetence [8]. In this setting, the use of neoadjuvant chemotherapy seems to be effective in shrinking the tumor to make it more amenable to surgery, with an overall response rate reported at around 70% [9]. Despite the fact that suboptimal response is apparently linked to a poor outcome [10], according to Cochrane’s meta-analysis (including 1078 patients [11] and other retrospective reviews and meta-analysis), neoadjuvant chemotherapy (NACT) improves oncological outcomes and reduces the need for post-operative therapy [12,13]. Furthermore, obstetrical outcomes seem superior when compared to upfront radical trachelectomy [14]. As for the choice of the proper therapeutic regime, many different schemes are being employed, with the three-drug combination of paclitaxel, ifosfamide and cisplatin being the most effective but also more toxic compared to paclitaxel and cisplatin [15]. Carboplatin has not proved to be inferior to cisplatin, is less toxic [16,17] and a weekly dose-dense regime with paclitaxel and carboplatin is associated with a good response and limited alopecia [18].

Results from recent studies, following the systematic review by Bentivegna and colleagues in 2016 [19], might have partially diminished the credibility of the oncological safety of neoadjuvant chemotherapy studies in IB2 cervical cancer (FIGO 2018) [20,21,22,23]. This review strives to provide more evidence in the feasibility, oncological and obstetric outcomes of neoadjuvant chemotherapy prior to fertility-sparing surgery in patients with a tumor diameter from 2 to 4 cm.

## 2. Materials and Methods

### 2.1. Search Strategy and Selection Criteria

**Figure 1:**
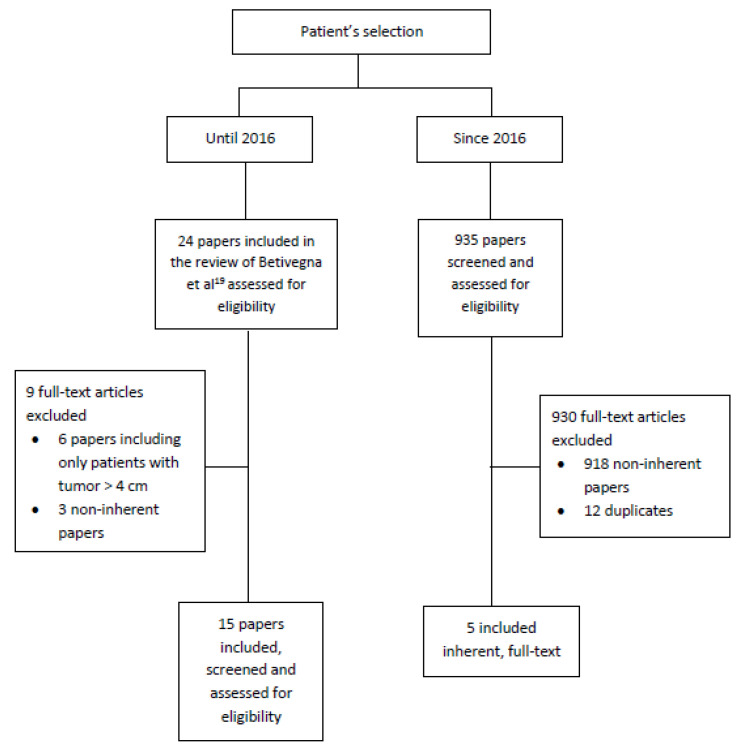
PRISMA 2009 flow diagram.

This systematic review was performed according to the Preferred Reporting Items for Systematic Reviews and Meta-Analyses (PRISMA) guidelines. Data were collected searching through PubMed, MEDLINE and references from the most relevant articles regarding this topic. Only articles in English and full text articles were included. We excluded papers on cervical cancer in pregnancy. In particular, we synthesized 15 papers on neoadjuvant chemotherapy followed by fertility-sparing surgery, from the systematic review regarding fertility-sparing surgery in cervical cancer by Bentivegna et al. in 2016 [19], from which we excluded some non-inherent papers. Moreover, we used the search terms “cervical cancer”, “fertility-sparing”, “neoadjuvant chemotherapy” and “radical trachelectomy” to complete the systematic review. Specifically, we conducted the following search: Search #1: (cervical cancer) AND (fertility-sparing) AND neoadjuvant chemotherapy: we found 57 articles and selected 6 articles published from July 2016 to April 2020; Search #2: (cervical cancer) AND (fertility-sparing): we found 331 articles and selected 9 articles published from July 2016 to April 2020; Search #3: (cervical cancer) AND (radical trachelectomy): we found 546 articles and selected 3 articles published from July 2016 to April 2020. In total, after removing duplicates, we screened and assessed 934 articles for eligibility and selected 5 inherent papers (Figure 1).

### 2.2. Data Collection and Analysis

Data were extracted from all series and case reports, in accordance with the selection criteria. We focused on records about tumor characteristics (size, histological subtype and grade lymph-vascular space invasion), chemotherapy regimens (type and number of cycles), the fertility-sparing surgical approach, oncological outcomes, pathological response to chemotherapy, rate and reasons for abandoning the fertility-sparing approach, type of recurrences, survival data and fertility outcomes (live births and failed attempts). Not all studies reported the depth of stromal invasion. All women received neoadjuvant chemotherapy consisting in a platinum-based treatment. The number of courses ranged from 1 to 4.

Pathological responses were defined as follows: optimal pathological response (OPR) that included a complete disappearance of tumor in the cervix with negative nodes (CR) or a residual disease with <3 mm stromal invasion including in situ carcinoma (PR1); and suboptimal response (SOR) that consisted of persistent residual disease with >3 mm stromal invasion on surgical specimen (PR2).

## 3. Results

We identified 20 series involving 114 patients with invasive cervical cancer FIGO 2018 IB2 as potential candidates for neoadjuvant chemotherapy prior to fertility-sparing surgery (Table 1).

The largest series included 14 women (articles range 1–14). Sixty-two patients presented squamous carcinoma (54.4%), twenty-three had adenocarcinoma (20.2%) and one had adeno-squamous (0.9%). Histology was not specified in 28 patients. Among the entire cohort, uterine conservation was feasible in 87 women. Overall, 27 women (23.3%) were not eligible for fertility-sparing surgery because of the persistence of tumor (*n* = 3), progression of disease after neoadjuvant chemotherapy (*n* = 3), lymph node positivity (*n* = 12), positive surgical margins (*n* = 6), for a personal decision not to complete the fertility-sparing approach (*n* = 1) and two for unknown reasons (Table 2).

Among the excluded, 16 underwent radical hysterectomy, 9 exclusively chemo-radiotherapy, whereas in 2 patients the type of definite treatment was not defined. Among the patients abandoning fertility-sparing surgery, 8 had adenocarcinomas and 9 had squamous carcinoma, while for the remaining 10 women, the histotype was not specified. Tumor size of the patients who abandoned fertility-sparing surgery was known for 17 women, and its median size was 27.9 mm. As for tumor grading, the data were available for 14 patients: 2 patients had grade 1, 3 patients had grade 2 and 7 had grade 3 tumors.

Among the different schedules of chemotherapy, the one most used was the three-drug combination including cisplatin, ifosfamide and paclitaxel (TIP), or the regimen containing epirubicin instead of ifosfamide (TEP) in the presence of adenocarcinoma histology (44%) (Table 1).

Eighty-seven women underwent conservative surgery after neoadjuvant chemotherapy. Seventy-seven patients underwent pelvic lymphadenectomy (PLND) prior to simple conization (*n* = 8; 9.2%), radical vaginal trachelectomy (VRT) (*n* = 35; 40.2%), radical abdominal trachelectomy (ART) (*n* = 10; 11.5%), radical laparoscopic trachelectomy (*n* = 8; 9.2%), simple vaginal trachelectomy (SVT) (*n* = 9; 10.3%) and either conization or simple vaginal trachelectomy (*n* = 7; 8.0%). Conization was only performed in one patient (1.1%), while nine patients underwent sentinel lymph node (SLN) procedure plus radical abdominal trachelectomy (10.3%). Only moderate toxic effects related to chemotherapy were reported: in particular, 14 patients suffered from Grade 3 hematological events (12.1%); there were three renal events (2.6%), one case of stroke (0.9%) and one case of hepatitis (0.9%). As for the surgery, very few intraoperative complications were described: one patient had a ureteral injury (1.1%) and one vessel injury occurred (1.1%). Overall, the rate of stenosis of the cervical canal was 25.3% (22/87), the majority of which were resolved with dilatation under anesthesia. Fourteen patients underwent ART (63.6%), 5 underwent SVT (22.7%) and 3 underwent cold knife conization (CKC) (13.6%). 

### 3.1. Pathological Responses after NACT

Overall, 53/87 (60.9%) patients achieved the optimal pathological response to neoadjuvant chemotherapy, among which 40 patients (46%) reached complete response and 13 (14.9%) optimal partial response (PR1). Instead, for 17 patients (19.5%), the pathological response was suboptimal (PR2). Pathological response was defined as tumor disappearance greater than 50% in 7 patients (8%) [34], whereas response was not described in 10 patients (11.5%). Chemotherapy is still ongoing in two patients (Table 1).

Pathological response differed on the basis of the regimen used. Among the 36 women treated with the three-drug combination of cisplatin, ifosfamide, paclitaxel (TIP) or epirubicin instead of ifosfamide (TEP), 20 patients had a complete response (55.6%), 9 had partial optimal response (PR1) (25.0%) and 7 showed PR2 (19.4%). Among the 12 patients treated with carboplatin and paclitaxel, 2 women had a complete response (16.6%), 1 patient had a PR2 (8.3%) while, for the 9 remaining patients, a pathological response was not reported (75%). Concerning the rest of the patients (*n* = 39) treated with other platinum-based regimens, an optimal pathological response was achieved in 23 patients (59.0%), including 18 complete responses (46.2%) and 5 partial optimal responses -PR1 (12.8%); 8 patients (20.5%) had a partial suboptimal response (PR2) and 1 had stable disease (2.6%). For seven patients, the authors reported a pathological response greater than 50% (17.9%) (Table 1).

### 3.2. Survival Data

Eight women who underwent fertility-sparing surgery recurred (9.2%) (Table 3). Five patients recurred locally on the cervix (62.5%). In two women the recurrence was loco-regional (one in the recto-vaginal septum, one in which the exact site was not specified) and, in one, the relapse was distant to the ovary (Table 3). In seven cases the tumor was squamous carcinoma (87.5%), while only one patient with recurrence had adenocarcinoma (12.5%). Among the eight women who recurred, five underwent simple vaginal trachelectomy after nodal staging and neoadjuvant chemotherapy with both ifosfamide plus cisplatin (4/5), or epirubicin plus the cisplatin combination (1/5). At final pathology, two achieved complete response, while one PR1 and two PR2 were observed. In three cases, after NACT with cisplatin plus paclitaxel (three-week or weekly schedule) a radical abdominal or vaginal trachelectomy was performed, achieving two suboptimal responses, including two PR2 and one stable disease.

### 3.3. Obstetrical Outcomes

Disease-free survival (DFS) was reported in 5/8 patients and was 7.4 months (range 3–17). At recurrence, two patients underwent surgery with adjuvant radiotherapy (with/without chemotherapy), whereas five women received concomitant chemo-radiotherapy. Regarding the latter two, in one patient, additional adjuvant chemotherapy was administered. One patient underwent a pelvectomy followed by concomitant chemo-radiotherapy. Only one woman underwent a radical hysterectomy. Four patients died of disease (4.6%), whereas two women are alive with no evidence of disease, and two are alive with tumor.

Obstetrical outcomes were reported for 42 women (Table 4). Twenty-one tried to conceive after the completion of fertility-sparing treatment (50.0%) and in all but three cases, pregnancy occurred spontaneously (85.7%). Four patients had one live birth at late preterm, among which two were caesarean section (CS); one had two live births at term; two had an early miscarriage; one had one ectopic pregnancy and one live birth at term with caesarean section; one had one early miscarriage and one preterm live birth; three had one live birth at term by caesarean section and one was also an early miscarriage. One woman has an ongoing pregnancy. The most common complications that occurred during pregnancy were intra-hepatic cholestasis of pregnancy (*n* = 1), gestational diabetes (*n* = 1), preterm premature rupture of the membranes (*n* = 2), premature contractions (*n* = 1) and vaginal bleeding (*n* = 1).

## 4. Discussion

The optimal management of cervical cancer patients with a tumor diameter between 2 and 4 cm (FIGO 2018 IB2), who wish to preserve fertility, is not well defined yet. International guidelines recommend a radical trachelectomy with pelvic node dissection as a fertility-sparing treatment for selected cases [3], but, in general, they advise fertility-sparing surgery in patients with cervical cancer ≥ 2 cm only as an experimental approach [38].

A recent study by Li et al. [39] reported very promising oncological outcomes in a large series of patients with FIGO 2018 stage IA1 with lymph-vascular space invasion to IB2 cervical carcinoma treated with radical abdominal trachelectomy; in particular, the recurrence and death rate among the 132 patients with tumors between 2 and 4 cm was 5.3% and 3.0%, respectively. Interestingly, the authors reported that adenosquamous histology was the only independent predictor of recurrence. Nevertheless, radical trachelectomy has been associated with infertility, adverse obstetrical outcomes (such as premature rupture of the membrane and premature labor) and urinary disorders [5,40]. For these reasons, neo-adjuvant chemotherapy followed by more conservative surgery (such as simple trachelectomy or conization) has been proposed as an alternative to radical trachelectomy in order to reduce tumor size and reduce unfavorable outcomes [14,19]. As shown in the findings from the present review, survival results from a simple trachelectomy or cervical conization after neoadjuvant chemotherapy were similar compared to non-fertility-sparing treatment of patients with the same tumor size. In particular, the recurrence and death rates from cervical cancer in patients undergoing neoadjuvant chemotherapy followed by a fertility-sparing treatment were 9.2% and 4.6%, respectively, which are comparable to the evidence in the literature reporting recurrence and death rates in patients treated with an upfront radical hysterectomy [2,41,42].

In their review, Bentivegna et al. compared 52 IB1 (FIGO 2008) patients with tumors greater than 2 cm, undergoing neoadjuvant chemotherapy and fertility-sparing surgery (FSS), with 209 patients with the same stage of disease, undergoing abdominal radical trachelectomy (ART). In the neoadjuvant chemotherapy group, the overall recurrence rate of 1B2 patients was 6% (3/52 patients), versus 7% (15 cases) in the radical abdominal trachelectomy group. Three patients (8%) had positive margins after neoadjuvant chemotherapy. The authors concluded that, according to overall recurrence, neoadjuvant chemotherapy prior to fertility-sparing surgery seemed interesting and acceptable in this group of patients [19]. However, 5 recurrences occurred among 26 women with Stage 1B2 disease included in 3 recently published studies (19.2%), increasing the overall rate of recurrence (Table 3) [20,22,23].

The driver of the relapse appeared to be the pathological response to chemotherapy, since 87% of the women who achieved an optimal pathological response (complete response or partial optimal response) did not experience any recurrence. Similarly, in the radical procedure group, no patients with documented optimal responses had a recurrence. Three out of fourteen patients with suboptimal response recurred (4.7%).

Therefore, response to neoadjuvant chemotherapy represents a crucial factor, not only in determining the feasibility of a subsequent fertility-sparing approach, but also as a prognostic factor. Studies in a no fertility-sparing setting showed that achieving an optimal response represents an important predictor of the survival in women with locally advanced cervical cancer (FIGO stage 2018 1B3-IIB) and a surrogate end point for treatment [43].

Histology of the tumor appears to be a significant predictive factor of response to NACT; indeed, in the SCC subtype, chemo prior to surgery was proved to be superior than in the adenocarcinoma or adenosquamous subtypes [44,45]. The first metanalysis investigating this topic, published by He et al. in 2014, concluded that histological type might be used to predict the long-term efficacy of NACT in cervical cancer, and that it was especially true for those with FIGO stages above IIB [46].

Various chemotherapy regimens have been associated with different degrees of pathological responses but with varying toxicity. A study performed on neo-adjuvant treatment of locally advanced squamous cell cervical cancer showed that the addition of ifosfamide to cisplatin and paclitaxel provided a higher pathological response rate but with worsened hematologic toxicity [15]. Moreover, some authors proposed weekly carboplatin/paclitaxel as an alternative to cisplatin/paclitaxel with a response rate comparable to the triplet but with reduced toxicity [18]. Nevertheless, as shown in Table 1, the chemotherapy regimens chosen as neoadjuvant chemotherapy prior to a fertility-sparing treatment have been extremely various throughout the years and across the different studies.

Concerning the extent of the cervical surgery in the case of optimal response, no randomized study compared the outcomes of conization versus simple trachelectomy. According to our review, most of the patients who recurred had previously undergone a simple or radical trachelectomy (Table 3). However, recurrences after conization have also been reported recently [20]. Nevertheless, we must acknowledge a potential selection bias, for which larger tumors with worse response to neoadjuvant chemotherapy have undergone more radical cervical surgery, but they also had an intrinsic higher risk of recurrence.

On the other hand, it is intuitive that the lower the radicality of the cervical procedure, the better the obstetrical outcome, particularly if neoadjuvant chemotherapy is administered instead of upfront surgery first [47]. Neoadjuvant chemotherapy followed by conization appears to be a promising alternative to upfront radical trachelectomy in terms of obstetrical results, as confirmed by the high pregnancy rate in this subgroup of patients (Table 4) [14]. This confirms that the main cause of infertility and obstetric failure after fertility-sparing surgery is related to cervical factors: mainly due to the lack of cervical mucus, cervical stenosis and a reduction in the length of the remaining cervix/uterine isthmus [5]. In a meta-analysis comparing conization with radical trachelectomy in the upfront setting, Zhang et al. [48] showed that miscarriage and preterm labor happened in 24.0% and 26.6%, with a pooled pregnancy rate of 20.5% in the radical trachelectomy group compared to the miscarriage and premature birth rate of conization of 14.8% and 6.8%, with a pregnancy incidence of 36.1%. In the present review, we showed that 85.7% of patients were able to conceive spontaneously, but 61.1% of patients experienced miscarriage or pre-term labor (Table 1 and Table 4). The pathogenesis of these adverse events was probably related to the shortened uterine cervical length. In the second trimester, these losses (and premature delivery) were also related to preterm premature rupture of membranes (pPROM), in a majority of cases, due to subclinical or clinical chorioamnionitis; the main explanation of this is linked to the absence of the cervix and the potential exposure to the vagina, thus increasing the risk of infection. For this reason, these patients carry the risk of first- and second-trimester miscarriage and preterm delivery. Given this higher rate of prematurity, the patient should be strongly advised to have the pregnancy, delivery, and follow-up in a maternity hospital, with personnel well trained in the management of high preterm and/or low birth weight infants.

Since many studies report a high incidence of lymph node metastases in 2–4 cm cervical tumors (18% in the study by Park et al. up to 45% according to Wethington et al.), performing a lymphadenectomy before FS surgery is undoubtedly necessary [2,38,49]. In general, according to NCCN guidelines, a pelvic lymphadenectomy is requested in FS surgery for cervical cancer in stage IA1 with LVSI, in stage IA2, IB1 and IB2 (in these last two stages paraortic lymphadenectomy is also an option) [50].

In the present study, all but one patient underwent retroperitoneal staging during surgery. In particular, in 25 patients, a lymphadenectomy was performed before NACT. Among our patients, in the totality of those who recurred, a systematic pelvic lymphadenectomy had been performed. Overall, among our patients, the rate of lymph node metastases was, however, lower if compared to the data in the literature (10.5% of women abandoned FS for nodal positivity).

Data are not mature yet to favor lymphadenectomy prior over after NACT in FS treatment. In the study by Rendón et al., recurrences occurred in up to 14% of patients who underwent a pre-NACT lymphadenectomy versus 12.8% in those with a post-NACT lymphadenectomy [51]. In our review, among patients who underwent retroperitoneal staging pre-NACT, two recurrences were reported, but such an approach was far less common (25 patients). Using NACT to treat metastatic and micro-metastatic surgery prior to FS might be a sensible option to downstage disease and allow more patients to proceed with fertility preservation [51].

Sentinel lymph node (SLN) mapping can be considered in all cases [50]. In the past few years, SLN biopsy has become more and more popular in early-stage cervical cancer in view of the optimal results in terms of accuracy and the reduction in lymphadenectomy-related complications [52,53]. Although randomized trials comparing SLN only with SLN and pelvic lymphadenectomy are still ongoing [54,55], multiple pieces of evidence show that SLN in cervical cancer provides important information about the possible presence of low-volume metastases and demonstrates a prognostic role in some studies [56,57], thus allowing further treatment tailoring. Few studies in the present series assessed SLN together with pelvic lymphadenectomy, and we believe that this may represent the future approach to retroperitoneal assessment, together with or replacing pelvic lymphadenectomy in patients undergoing neoadjuvant chemotherapy and fertility-sparing surgery.

Data about LVSI are, unfortunately, only partially known among patients included in the studies and no firm conclusions about characteristics of recurrences can, therefore, be driven. As far as we know, among patients who abandoned FS, two had positive LVSI and, among them, one was in the presence of massive persistence of disease after NACT [27] and the other one was because of nodal positivity [21]. On the other hand, among patients who underwent FS and recurred, two out of three patients in the study by Robova et al. [35] had positive LVSI, and the status was unknown for the third one; two out of two recurrences in the study of Slama et al. had positive LVSI (100%) [20], while no data were reported for the patient in the study by Tesfai et al. [22] nor for the two in the study by Marchiolè et al. [23].

According to the literature, LVSI is one of the most important prognostic factors in early-stage cervical cancer and represents a criterion used to stratify global patients’ risk [38]. In 1990, the GOG-49 study concluded that 3-y DFS was also influenced, apart from tumor size and stromal invasion depth, by LVSI status [58]. Recently, Ronsini et al. proposed a semi-quantitative analysis of LVSI (absent, focal and diffuse), concluding that LVSI is significantly associated with a higher risk for lymph node metastasis and that diffuse LVSI correlates with a worse DFS than focal or absent and a higher risk for nodal and distant recurrences [59].

Therefore, the presence of positive LVSI represents and independent prognostics factor for survival, and this should be considered even after NACT with fertility-sparing intents to guide the decision-making process for this subgroup of women.

One last point of discussion should be presented regarding the surgical approach to the cervical procedure. It is well known that a randomized trial demonstrated a higher rate of recurrence and death in patients undergoing radical hysterectomy with a minimally invasive approach, compared to the open approach (Laparoscopic Approach to Cervical Cancer, LACC trial) [42]. Even though the causes of such results are still not completely clear [60,61,62,63], different factors such as peritoneal tumor contamination, use of a manipulator and extent of radicality have been advocated [64]. In this context, fertility-sparing surgery after neoadjuvant chemotherapy can be advocated in cases undergoing radical trachelectomy with an open or minimally invasive approach, while for conization and simple trachelectomy, this should not be the issue. Regarding a radical trachelectomy, a recent study on the robot-assisted laparoscopic trachelectomy demonstrated promising results in terms of oncological (4% of patients recurred) and obstetrical (81% of patients conceived and 94% delivered in the third trimester) outcomes [65].

**Figure 2:**
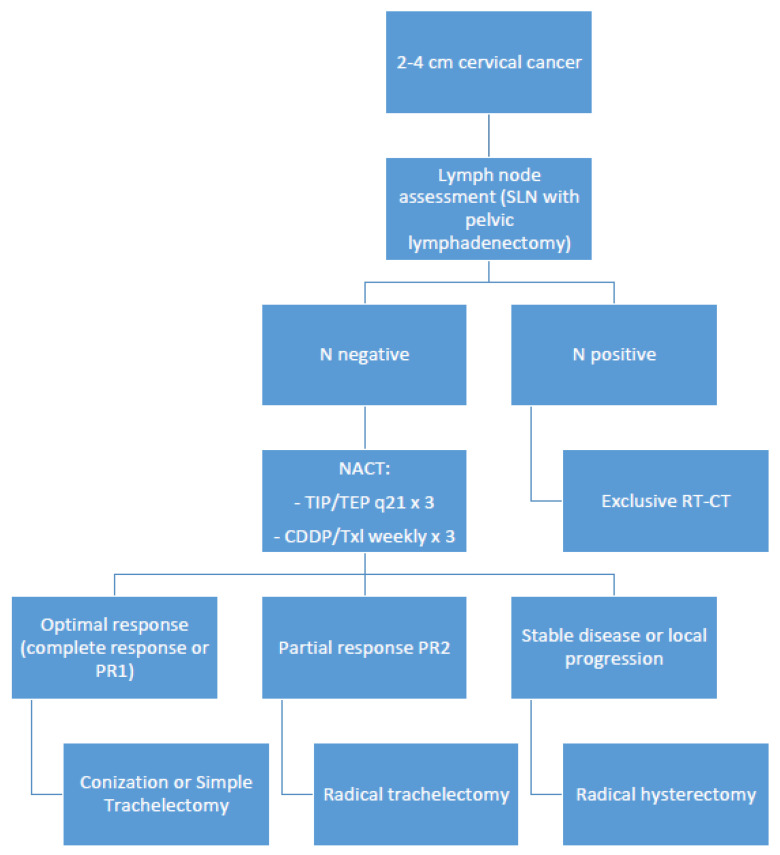
Decision-making process algorithm for women with IB2 cervical cancer. SLN = sentinel lymph node. N = lymph nodes. NACT = neoadjuvant chemotherapy. RT-CT = radiochemotherapy. TIP = cisplatin-paclitaxel-ifosfamide. TEP = cisplatin-paclitaxel-epirubicin. CDDP/TXL = cisplatinum-paclitaxel.

Based on the present systematic review, it appears evident that the “one-size-fits-all” concept cannot be applied to patients with FIGO 2018 1B2 cervical cancer who wish to preserve fertility. Therefore, in view of the present era of personalized medicine, we have developed an algorithm that could be helpful in the decision-making process for these young women who find themselves between the hammer and the anvil. In the presence of a suboptimal response, a radical trachelectomy should be considered and offered as an option to women highly motivated to preserve fertility (Figure 2).

## 5. Conclusions

At the end of our review process, we want to underline the weaknesses of the available literature regarding this issue, particularly in relation to treatment selection bias for these patients who have decided to preserve fertility, in spite of the presence of cervical cancer. To date, the evidence is too scanty to draw firm conclusions. However, from the studies where neoadjuvant chemotherapy to fertility-sparing surgery was offered, our revision has shown that a suboptimal response to neoadjuvant chemotherapy seems to be an independent prognostic factor in poorer survival.

Therefore, a careful selection of subjects and the surgical approach after neoadjuvant chemotherapy remains crucial in balancing the obstetric outcomes, with the risk of non-responsiveness that impairs both obstetric and oncological outcomes. The ongoing CONTESSA and IRTA studies are investigating these debated issues.

The scientific community awaits with interest the results of the international collaboration of the IRTA study [66], which is assessing the oncological outcomes of patients who underwent trachelectomy with the open versus the minimally invasive approach, and the prospective phase II single arm CONTESSA trial [67], which is addressing the safety of neoadjuvant chemotherapy followed by fertility-sparing surgery in young women with International Federation of Gynecology and Obstetrics FIGO 2018 stage IB2 cervical cancer, who wish to preserve fertility. In the meantime, neoadjuvant chemotherapy prior to fertility-sparing surgery should be carefully discussed with women and their partners to underline and clearly state the risks of such an approach, balancing the best fertility results with the best chance of a cure as well as the risk of recurrence and survival. Therefore, in this context, the adequate counselling of women remains essential in the decision-making process for a fertility-sparing approach.

## Figures and Tables

**Table 1:** Studies of neoadjuvant chemotherapy with conservative management for stage IB2 cervical cancer.

Study	N pts Underwent FSS	Histological Subtype	Chemo Regimen	Surgery after NACT	Response	Recurrence (Yes/NO)	Site of Recurrence	Obstetrical Outcome	Obstetrical Complications	Status	Reference
Wang et al., 2013	2	2 SCC	1 Cisplatin, fluorouracil; 1 bleomycin, cisplatin	Pelvic LND + VRT	1 CR, 1 PR2	No recurrence (median 82 months)	-	0 pregnancies in 0 attempting	NA	NED	[24]
Kobayashi et al., 2006	1	1 SCC	Cisplatin, bleomycin, vincristine, mitomycin C	CKC	CR	No recurrence (median 48 months)	-	1 livebirth of 1 attempting	None	NED	[25]
Plante et al., 2006	3	3 SCC	Cisplatin, paclitaxel, ifosfamide	Pelvic LND + VRT	CR	No recurrence (median 60 months)	-	3 live births and 1 T1 loss in 3 attempting	None	NED	[26]
Maneo et al., 2008	6	3 SCC, 3 ADK	3 Cisplatin, paclitaxel, ifosfamide; 3 Cisplatin, paclitaxel, epirubicin	Pelvic LND + CKC	3 CR, 3 PR1	No recurrence (median 69 months)	-	NA	NA	NED	[27]
Liu et al., 2008	1	1 SCC	Cisplatin, bleomycin	Pelvic LND + ART	PR2	NA	NA	1 livebirth of 1 attempting	Intrahepatic cholestasis of pregnancy	NA	[28]
Marchiolè et al., 2011	2	2 SCC	Cisplatin, paclitaxel, ifosfamide	Pelvic LND + VRT	1 CR, 1 PR2	No recurrence (median 25 months)	-	0 pregnancies in 0 attempting	NA	NED	[29]
Singh et al., 2011	1	1 ADK *	Carboplatin, paclitaxel	Pelvic LND + VRT	PR2	No recurrence (median 14 months)	-	NA	NA	NED	[30]
Plante et al., 2011	1		Cisplatin, gemcitabine	Pelvic LND + VRT	CR	No recurrence	-	0 pregnancies in 0 attempting	None	NED	[5]
Vercellino et al., 2012	4	2 SCC, 2 ADK	4 Cisplatin, paclitaxel, ifosfamide;	VRT (LND prior to CT)	3 CR, 1 NA	No recurrence (median 30 months)	-	0 pregnancies	NA	NED	[31]
Tsubamoto et al., 2012	1	1 SCC	Irinotecan, intrauterine artery cisplatin	Pelvic LND + SVT	CR	No recurrence (median 65 months)	-	0 pregnancies in 0 attempting	NA	NED	[32]
Lanowska et al. 2014	12 (+2 ongoing)	5 (+2) SCC, 6 ADK, 1 ADS	Cisplatin, paclitaxel, ifosfamide	VRT (P+PA LND prior to CT)	7 CR, 2 PR1, 3 PR2, 2 pz ongoing	No recurrence (median 23 months)	-	7 pregnancies in 5 pts: 4 live births, 1 ongoing pregnancy, 2 T1 loss	1 GD, 1 pPROM, 1 premature contractions, 1 vaginal bleeding	NED	[33]
Lu et al., 2014	7	7 SSC	Intra-arterial cisplatin, bleomycin, mitomycin	PLDN + laparoscopic radical trachelectomy	7 Response > 50%	No recurrence (median 66 months)	-	1 live birth and 1 T1 loss in 4 attempting	1pPROM	NED	[34]
Robova et al., 2014	8		Cisplatin, ifosfamide; cisplatin, doxorubicin	Pelvic LND + SVT	5 CR, 1 PR1, 2 PR2	2 Local recurrences, 1 ovarian recurrence, 2 died from disease (median 42 months)	2 local, 1 ovarian	NA	NA	2 died from disease, 6 NED	[35]
Saadi et al., 2015	1	1 SCC	Cisplatin, fluorouracil, ifosfamide	PLDN + Laparoscopic radical trachelectomy	CR	No recurrence (median 9 months)	-	NA	NA	NED	[36]
Salihi et al., 2015	2	1 SCC, 1 ADK	1 Cisplatin, paclitaxel, ifosfamide, 3 weekly carboplatin, paclitaxel	PLDN + CKC	2 CR	No recurrence (median 58 months)	-	1 live birth in 2 attempting	None	NED	[18]
Slama et al., 2016	7 (IB1 + IB2)	7 SCC	Cisplatin, ifosfamide	CKC/SVT (P LND prior to CT)	7 CR	2 local recurrences, 1 died from disease (median 23 months)	local	NA	NA	1 died from disease, 1 NED	[20]
Tesfai, 2020	9	9 SCC	Wk Carboplatin, paclitaxel	PLND+ ART	1 CR,4 PR1, 4PR2	2 recurrence (3 and 17 months)	loco-regional	NA	NA	1 died from disease, 8 NED	[22]
Marchiolè et al., 2018	10	8 SCC, 2 ADK	8 TIP, 1 TEP, 1 cisplatin, paclitaxel	PLND + VRT	3 CR, 3 PR1, 3 PR2, 1 SD	2 local recurrences (median 79 months)	local	1 live birth, 1 T1 loss in 3 attempting	None	2 alive with metastatic disease	[23]
Okugawa et al., 2020	9		Carboplatin, paclitaxel	SLN +ART		No recurrences (median 72 months)	-	NA	NA	NED	[37]
Bogani et al., 2019	0/2	-	N.S.	(CKC)		(No recurrence)	-	Fertility-sparing treatment abandoned	None	-	[21]

Legend. N: number; CT: chemotherapy; NAC = neoadjuvant chemotherapy; SCC = squamous cervical carcinoma; ADK = adenocarcinoma; ADS = adenosquamous carcinoma; CKC = cold knife conization; CR = complete response; PR1 = optimal partial response (residual with <3 mm stromal invasion); PR2 = suboptimal partial response (residual with >3 mm stromal invasion); SD = stable disease; LND = lymphadenectomy; PLDN = pelvic lymphadenectomy; P + PA = pelvic + para aortic; SLN = sentinel lymph node biopsy; VRT = vaginal radical trachelectomy; ART = abdominal radical trachelectomy; SVT = simple vaginal trachelectomy; T1 = first trimester; pPROM = preterm premature rupture of membranes; GD = gestational diabetes; Wk = weekly; TIP = cisplatin- ifosfamide-paclitaxel; TEP = cisplatin- epirubicin-paclitaxel; NS = not specified; NA = not applicable; * = clear cell.

**Table 2:** Characteristics of patients abandoning the fertility-sparing approach after neoadjuvant chemotherapy.

Study	N pts Abandoning FFS	Histological Subtype	Tumor Size	Grade	LVSI	RT	Nodal Status	Reason for Abandoning	Subsequent Treatment	Status	Reference
Maneo et al., 2008	2/8	2 ADK	30 mm, 20 mm	1 G1, 1 G3	1 No, 1 Yes	1 in situ, 1 massive involvement	2 Negative	1 personal reason, 1 massive persistence	2 RH	2 NED	[27]
Robova et al., 2014	8	NA	NA	NA	NA	NA	NA	6 positive margins, 2 positive lymph nodes	8 RH	NA	[35]
Vercellino et al., 2012	8/12	4 SCC, 4 ADK	21 mm, 22 mm, 25 mm (3), 30 mm, 35 mm, 40 mm	5 G3, 3 G2	NA	-	7 pelvic positivity; 1 aortic positivity	8 lymph node positivity	8 CRT	6 NED, 2 DOD	[31]
Salihi et al., 2015	2/4	1 SCC, 1 ADK	20 mm, 25 mm	1 G1, 1 G3	NA	23 mm, 30 mm	2 Negative	1 PR, 1 PD	2 RH	1 NED, 1 adjuvant RCT *	[18]
Slama et al., 2016	2/9 (IB1 + IB2)	2 SCC	20 mm, 40 mm	NA	NA	-	-	PD during NACT	-	-	[20]
Bogani et al., 2019	2/2	2 SCC	20 mm, 40 mm	NA	1 Yes, 1 No	1 NA, 1 SD	1 IIIC1	1 positive node (MM), 1 tumor size	1 RH, 1 RCT	2 NED	[21]
Tesfai et al., 2020	1/10	ADK	40 mm	NA	NA	>3 mm stromal invasion	1/23	Intraoperative pelvic node positivity	RH	DOD (after 3 month)	[22]
Okugawa et al., 2020	2	NA	NA	NA	NA	NA	NA	NA	2 RH	NA	[37]

Legend. NACT = neoadjuvant chemotherapy; LVSI = lymph-vascular space invasion; SCC = squamous cervical carcinoma; ADK = adenocarcinoma; ADS = adenosquamous carcinoma; CR = complete response; PR = partial response; SD = stable disease.; PD = progressive disease; RT = residual tumor; NED = no evidence of disease; DOD = died of disease; RH: radical hysterectomy; RCT = radio-chemotherapy; MM = micrometastasis; OS = overall survival; NA = not available; *: ongoing.

**Table 3:** Analysis of cases of recurrences.

Study	Pts Recurred	Histology	Tumor Size (mm)	NACT Regimen	FS Surgery	Pathological Response	RT (mm)	Type of Recurrence	DFI (Months)	Treatment at Recurrence	Status	Reference
Robova et al., 2014	3/8	SCC	24	IP	PLND + SVT	PR2	13	Distant (ovary)	NA	Surgery, adjuvant RT + CHT	DOD	[35]
		SCC	30	IP	PLND + SVT	PR1	2	Local (cervix)	NA	CHRT	DOD	
		ADK	25	EP	PLND + SVT	PR2	10	Local (cervix)	NA	Surgery, adjuvant RT	NED	
Slama et al., 2016	2/7	SCC	NA	IP	PLND + SVT	CR	0	Local (cervix)	6	RH	NED	[20]
		SCC	NA	IP	PLND + SVT	CR	0	Local (cervix)	7	CHRT, CHT	DOD	
Tesfai et al., 2020	1/9	SCC	35	wk CT	PLND + ART	PR2	NA	Loco-regional	17	CHRT	DOD	[22]
Marchiolè et al., 2018	2/10	SCC	30	CT	PLND + VRT	SD	30	Pelvic (central)	4	CHRT, pelvectomy	AWT	[23]
		SSC	34	TIP	PLND + VRT	PR2	15	Local (RVS)	3	CHRT	AWT	

Legend. NACT: neoadjuvant chemotherapy; FS: fertility-sparing; DFI = disease free interval; wk = weekly; RT = radiotherapy; CHT = chemotherapy; CHRT = chemoradiotherapy; IP= cisplatin, ifosfamide; EP = cisplatin, doxorubicin; CT = cisplatin, paclitaxel; TIP = cisplatin-paclitaxel-ifosfamid; RH = radical hysterectomy; SCC = squamous cervical carcinoma; ADK = adenocarcinoma; PR1 = partial response (disease with <3 mm stromal invasion); PR2 = partial response (disease with >3 mm stromal invasion); PLND = pelvic lymphadenectomy; VRT = vaginal radical trachelectomy; ART = abdominal radical trachelectomy; SVT = simple vaginal; RT = residual tumor; NED = not evidence of disease; AWT = alive with tumor; DOD: Died of disease; RVS = Recto-vaginal space; NA = not available.

**Table 4:** Obstetrical outcomes among women who completed the fertility-sparing approach.

Study	Patients Attempting Pregnancy (%)	Spontaneous Conception	Pregnancy Outcomes	Complications during Pregnancy	Reference
Kobayashi et al., 2006	1/1 (100)	Yes	1 pt with live birth (vaginally) at 36 w	None	[25]
Plante et al., 2006 + 2011	3/4 (75)	3 yes, 1 no (Clomid + IUI)	1 pt with 2 live births at term; 1 pt with 1 live birth at term; 1 miscarriage at 8 w	None	[5,26]
Liu et al., 2008	1/1 (100)	Yes	1 with live birth at 35 w (CS)	Intrahepatic cholestasis of pregnancy	[28]
Marchiolè et al., 2011	0/2 (0)	-	-	-	[29]
Tsubamoto et al., 2012	0/1 (0)	-	-	-	[32]
Wang et al., 2013	0/2 (0)	-	-	-	[24]
Lanowska et al. 2014	7/12 (58.3)	Yes *	1 pt has ectopic pregnancy and livebirth (CS) at 38 w; 1 pt with ongoing pregnancy; 1 pt with early miscarriage and live birth (CS) at 31 w; 1 pt with live birth (CS) at 33 w; 1 pt with live birth (CS) at 37 w	1 GD, 1 pPROM, 1 premature contraction, 1 vaginal bleeding	[33]
Lu et al., 2014	4/7 (57.1)	Yes	1 pt with early miscarriage, 1 pt with live birth (CS) at 32 w	1 pPROM	[34]
Salihi et al., 2015	2/2 (100)	Yes	1 pt with live birth (vaginally) at 37 w	None	[18]
Marchiolè et al., 2018	3/10 (30)	Yes	1 pt with live birth (CS) at 37 w and with early miscarriage	None	[23]

Legend. Pt = patient; IUI = intrauterine insemination; W = weeks of gestation; CS = caesarian section; pPROM = pre-term premature rupture of the membranes; GD = gestational diabetes; * = 2 patients underwent fertility treatment after trachelectomy; None = not available.
